# Glycosylated Cathepsin V Serves as a Prognostic Marker in Lung Cancer

**DOI:** 10.3389/fonc.2022.876245

**Published:** 2022-04-13

**Authors:** Lewei Yang, Qi Zeng, Yun Deng, Yeqing Qiu, Wei Yao, Yifeng Liao

**Affiliations:** Department of Oncology, The fifth Affiliated Hospital of Sun Yat-sen University, Zhuhai, China

**Keywords:** *CTSV*, lung cancer, metastasis, glycosylation, prognosis

## Abstract

Dysregulation of cysteine cathepsin protease activity is pivotal in tumorigenic transformation. However, the role of cathepsin protease in lung cancer remains unknown. Here, we analyzed GEO database and found that lung cancer presented high expression of cathepsin V (*CTSV*). We then performed immunohistochemistry assay in 73 paired lung cancer tissues and normal lung tissues and confirmed that *CTSV* is overexpressed in lung cancer and correlates with poor prognosis. The mass spectrometry experiment showed that the N-glycosylation locus of *CTSV* are N221 and N292, glycosylated *CTSV* (band 43 kDa) was particularly expressed in lung cancer samples and correlated with lymph node metastasis. Mechanistic studies showed that only glycosylated *CTSV* (43-kDa band) are secreted to extracellular matrix (ECM) and promoted the metastasis of lung cancer. Importantly, the Elisa detection in serum of 12 lung cancer patients and 12 healthy donors showed that the level of *CTSV* in serum distinguished lung cancer patients from healthy donors. Together, our findings reveal the clinical relevance of *CTSV* glycosylation and *CTSV* drives the metastasis of lung cancer, suggesting that the glycosylated *CTSV* in serum is a promising biomarker for lung cancer.

## Introduction

Lung cancer is the leading cause of cancer-associated mortality worldwide (1). The high lethality rate observed in lung cancer patients is related to the common diagnosis of advanced stage disease, which hinders curative treatment and indicates poor 5-year survival. Metastasis has been proven to be the primary cause of cancer-related death among lung cancer patients (2, 3). Known protooncogenes of lung cancer include *EGFR, HER2, HER3, AKL/EML4, KRAS, BRAF, MET, NRAS, PIK3CA* and *ROS1*, among others (4, 5). Lung cancer-related tumor suppressors include *RBM5, FUS1, TP53, LKB1, SNPs, APC*, etc. The upregulation of oncogenes and the mutation of tumor suppressor genes may both involved in the progression of lung cancer (6). In recent years, targeted therapeutics in the treatment of lung cancer would be a substantial advance, as such events represent new targets for promising therapeutic intervention (7–10). Consequently, understanding the molecular mechanisms of lung cancer pathogenesis and identifying potential therapeutic targets for the clinical management of lung cancer are imminent research objectives.

Recently study showed that released cathepsins increases tumor cell invasion and metastasis in lung cancer (11). CTSS degradation of nidogen-1 is strongly associated with NSCLC (12). CTSF was found to be downregulated in NSCLC samples and overexpression of CTSF was correlated with favorable prognosis of NSCLC (13). Also, CTSB was proved to be closely related to the pathogenesis of lung cancer in a mouse model (14). The regulation of cathepsin C and cathepsin H (*CTSC* and *CTSH*) can cause a split anergic state in NK cells (15). The cysteine proteinase cathepsin O (*CTSO*) decreases the protein levels of *BRCA1* and *ZNF423* by proteinase-mediated degradation to regulate the tamoxifen sensitivity of breast cancer (16). Mutation of cathepsin F (*CTSF*) is correlated with type B Kufs disease and Alzheimer’s disease (17, 18). Cathepsin W (*CTSW*) is an important host factor for the entry of influenza A virus into target cells from late endosomes (19). Cathepsin V (*CTSV*) has been shown to be a tumor metastasis-associated protease in colorectal cancer, breast carcinoma and thymic epithelial tumors and is correlated with poor outcomes (20–23). Yuki Niwa et al. demonstrated that the N-glycosylation of *CTSV* is crucial for its transportation to lysosomes and secretion (24). However, the detailed picture of the downstream molecular mechanism of *CTSV* is still unclear. It is of great importance to understand the crucial roles and individual functions of *CTSV* in disease from the perspectives of both basic science and pharmacological targeting.

In this study, we reported that the overexpression of *CTSV* in lung cancer patients are positively correlated with poor overall survival. *CTSV* was glycosylated at N221 and N292 sites and the glycosylation of *CTSV* is correlated with lymph node metastasis. Notably, serum (secreted) *CTSV* distinguished lung cancer patients from healthy donors, and glycosylated *CTSV* determined its secretion and pro-metastatic role in lung cancer. Our study reveals glycosylated *CTSV* holds promise for providing effective prognostic biomarker for lung cancer patients.

## Methods and Materials

### Human Tissue Samples

73 fresh-frozen paired samples of primary lung cancer tissues and normal lung tissues were obtained from the Oncology Department at The Fifth Affiliated Hospital of Sun Yat-sen University. All human sample studies were reviewed and approved by the Ethics Committee of Sun Yat-sen University Cancer Center (GZR2020-154), and informed written consent was obtained from all donors.

### Cell Culture and Transfection

All cell lines A-549 (RRID : CVCL_0023), NCI-H1975 (RRID : CVCL_1511), NCI-H292 (RRID : CVCL_0455), HEK293T (RRID : CVCL_0063), NCI-H1993 (RRID : CVCL_1512) were purchase from ATCC and identified by short tandem repeat (STR) profiles from the China Center For Type Culture Collection (CCTCC, Wuhan University, China). All experiments were performed with mycoplasma-free cells. Cells were cultured with Dulbecco’s modified Eagle’s medium media (Thermo Scientific, USA) with 10% fetal bovine serum (FBS, Thermo Scientific, USA). HEK-293T cells were cultured in a six-well plate with complete culture medium the day before and transfected when the cell confluence was approximately 90%. We used a plasmid lentiviral packaging system, and the two packaging plasmids psPAX2 and PMD2.G (Thermo Scientific, USA) and the target gene were mixed at 3 μg:2 μg:1 μg. Then, polyethylenimine (PEI) was added to a quarter of the plasmid. Finally, 200 μL Opti-MEM medium was added to the mixture, and then the mixture was evenly added to the cells. After 6-8 hours, the solution was changed to complete medium. After 48 hours, the cell culture supernatant was collected and filtered with a 0.4-μm filter membrane to obtain virus solution.

### Migration and Invasion Assays

The indicated lung cancer cell migration and invasion ability were detected by Transwell assay using 24-well Boyden chambers (BD Inc., USA) with 8-μm pores coated with (invasion) or without (migration) Matrigel. We seeded 5×10^4^ A549 cells or 1×10^5^ (NCI-H1975 and NCI-H292) cells per well on the Transwell inserts and incubated them in 300 μL of serum-free DMEM at 37°C in the top chambers for 12 hours and 24 hours, whereas DMEM containing 20% FBS was added to the lower chamber. Cells that traversed the inserts to the bottom chamber surface were fixed, stained and observed under phase-contrast microscopy.

### Western Blotting

Briefly, cells were collected and analyzed in lysis buffer (50 mM Tris-HCl (pH 7.4), 150 mM NaCl, 1% NP-40, 0.1% SDS) supplemented with 1% protease inhibitor (Sigma-Aldrich, USA) after being gently washed 3 times with cold PBS, and then the lysate supernatant was obtained after centrifugation at 13,000 ×g and 4°C for 20 min. Protein (20 μg) was loaded and separated on 10% SDS-PAGE minigels, transferred onto PVDF membranes (Millipore, USA) and blocked with 5% blocking buffer for 1 hour at room temperature. The PVDF membranes were incubated with primary antibody overnight at 4°C with constant shaking, followed by incubation with horseradish peroxidase-conjugated secondary antibody (Promega, USA) for 1 hour at room temperature. The proteins were detected with an enhanced chemiluminescence (ECL) system (Bio-Rad, USA).

### Protein Mass Spectrometry Analysis

Briefly, A549 and NCI-H1975 cells were transfected to express Flag-tagged *CTSV*. The cells were then lysed in NETN buffer containing 15 mmol/L NaF, 60 mmol/L β-glycerophosphate, and 1 mg/mL each of pepstatin A and aprotinin. The debris was removed and incubated with Flag-conjugated beads at 4°C for 4 hours. Then, the beads were washed with NETN buffer 5 times, the bound proteins were analyzed by SDS-PAGE, and protein mass spectrometry was performed by PTM BioLabs. The immunocomplexes were then washed 4 times with NETN buffer and determined by SDS-PAGE and Western blot.

### Immunohistochemistry Staining

Lung cancer tissues were fixed in 4% paraformaldehyde for 16 hours at room temperature and then dehydrated in descending concentrations of ethanol. The tumor sections were incubated in 0.3% H_2_O_2_ solution at room temperature for 20 min and washed with PBS 3 times. Then, the cells were probed with monoclonal anti-*CTSV* (1:200) at 4°C overnight in a humidified container. After washing with PBS 3 times, the tissue sections were treated with goat anti-rabbit at room temperature for 2 hours. Streptavidin/peroxidase complex and diaminobenzidine were used for immunostaining, and hematoxylin was used for counterstaining.

### Antibodies

Antibodies specific for *CTSV* (WB: Abcam Cat#ab166894, IHC: Theremofish Cat#PA5-47061, ELISA: Theremofish, Cat#PA5-112393, RRID : AB_2261304), Flag-Tag (Cell Signaling, Cat#14793S, RRID : AB_2797401), HA-Tag (Cell Signaling, Cat#3724S, RRID : AB_391833), Tubulin (Genetex, Cat#GTX112141, RRID : AB_1157911) HSP70 (Abcam, Cat#ab2787, RRID : AB_1874830) and GAPDH (Genetex, Cat#GTX100118, RRID : AB_2617427) were purchased from the indicated companies.

### Concentration Medium Obtaining

The condition medium from indicated cultured cells were collected, and centrifuge at a centrifugal force of 2,000g per minute for 20 minutes, collected the supernatant for centrifuge at a centrifugal force of 10,000g per minute for 1 hour, collected the supernatant and repeated the centrifugation with 100,000g for 1 hour. Finally subjected the supernatant to concentration column and obtained that concentrated medium(CM).

### Statistical Analysis

All data were obtained from two or three independent experiments and are presented as the mean ± SD. We conducted the analysis in version 7.00 of GraphPad Prism using the unpaired, two-tailed Student’s t-test module, where ns represents no statistical significance, * represents *P* value<0.05, ** represents *P* value<0.01, *** represents *P* value<0.001, and **** represents *P* value<0.0001. Graphs were drawn with GraphPad Prism software. Kaplan-Meier survival analyses were used to compare survival among lung cancer patients based on *CTSV* expression. Western blot bands were quantified with Image-Pro Plus, and the results were normalized to those for the control GAPDH. Statistically significance was defined as *P*<0.05.

## Results

### *CTSV* Is Upregulated in Lung Cancer and Correlated With Poor Survival

**Figure 1:**
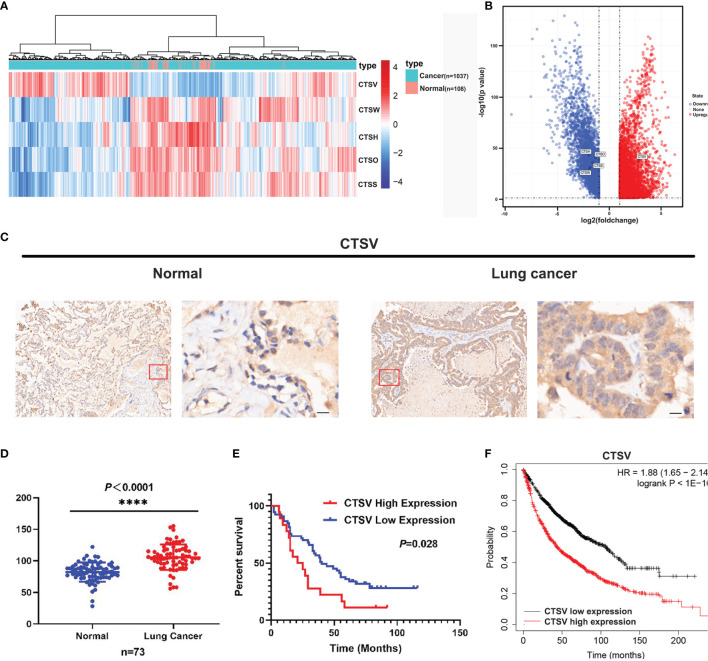
Lung cancer patients presented with high *CTSV* expression. **(A)**, **(B)** Analysis of mRNA expression of cysteine cathepsin family members (11 members) in lung cancer tissue and normal tissue from the TCGA database. **(C)** Representative images of CTSV IHC staining of lung cancer tissue and normal tissue. Scale bars, 50 μm. **(D)** Expression of CTSV in 73 paired lung cancer normal tissue samples. **(E)** Kaplan-Meier survival curves based on CTSV expression in lung cancer tissues. **(F)** Analysis of lung cancer patients’ overall survival in the Kaplan-Meier Plotter database. ****P < 0.0001.

Cysteine cathepsins are pivotal in disease development and progression; they have been reported to be overexpressed in a number of cancers and to lead to increased cancer cell invasion and metastasis (25–27), however, many facets of *CTSV* activity in lung cancer remain uncharacterized. To explore the role that the cysteine cathepsin family plays in lung cancer pathogenesis, we investigated The Cancer Genome Atlas (TCGA) and Gene Expression Omnibus (GEO) datasets and compared the gene expression of cathepsins family (*CTSB, CTSC, CTSF, CTSH, CTSK, CTSL, CTSO, CTSS, CTSV, CTSW* and *CTSX*) changes in lung cancer tissues and normal tissues. The RNA sequence data from the TCGA demonstrated that the expression of *CTSW, CTSH, CTSO* and *CTSS* was downregulated in lung cancer tissues; notably, only *CTSV* was found upregulated in lung cancer tissues (n=1037), approximately 2.6-fold higher than that in normal tissues (n=108) (**Figures 1A, B**), and no significant changes were observed in the remaining cathepsin family members. In addition, analysis of GEO datasets (GSE31210, GSE19188 and GSE3269) showed that lung cancer exhibits high *CTSV* levels (**Supplementary Figures S1A–C**). Next, we performed microarray gene expression profile analysis of lung cancer tissues from patients (n=73) with newly diagnosed lung cancer with paired adjacent normal tissues. Immunohistochemical staining of *CTSV* showed that *CTSV* expression in lung tumor tissues was higher than that in normal tissues (*P*<0.0001) (**Figures 1C, D**). We then analyzed the association of *CTSV* expression with the prognosis of 73 lung cancer patients. Our Kaplan-Meier analysis revealed that high expression of *CTSV* were correlated with poor overall survival (*P*=0.028) (**Figure 1E**). Further exploration of the Kaplan-Meier Plotter database (28) showed that elevated *CTSV* expression was correlated with poor survival in cancer patients (**Figure 1F**). Collectively, these results showed that *CTSV* is upregulated in lung cancer and is correlated with poor overall survival, implying the oncogenic role of *CTSV*.

### *CTSV* Is Glycosylated in Lung Cancer

**Figure 2:**
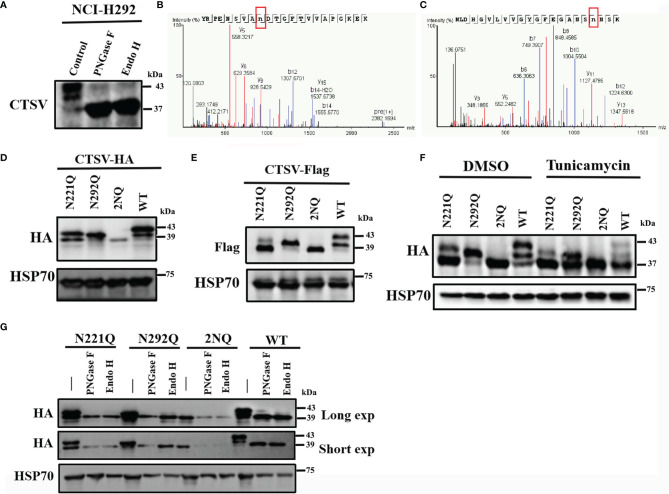
Identification of the glycosylation locus *CTSV*. **(A)** NCI-H292 cells lysates treated with PNGase F and Endo H were analyzed by Western blotting. **(B, C)** LC-MS/MS of the CTSV N221 and N292 glycosylation sites. **(D, E)** Cells transfected with the indicated plasmids with N221, N292 or both mutated sites were analyzed by Western blotting. **(F)** The indicated cells with N221Q, N292Q or both mutated sites were incubated with DMSO and tunicamycin (100ng/mL) and then analyzed by Western blotting. **(G)** Western blot analysis of the indicated stable cells lysates treated with PNGase F and Endo H with a long exposure time (60 sec) and short exposure time (20 sec).

While detecting the protein expression of *CTSV* in cancer cell lines, we noticed that the *CTSV* detection by Western blotting revealed more than one band, and the majority of the protein was found at 43 kDa. To determine whether the higher band is responsible for *CTSV* glycosylation, we treated NCI-H292 cells lysates with peptide-N-glycosidase F (PNGase F) and endoglycosidase (Endo H) to remove the structure of N-glycan and then analyzed the cell lysates by Western blotting. A significant reduction in 39- and 43-kDa *CTSV* and a remarkable increase in 37-kDa *CTSV* were observed (**Figure 2A**). The results generally revealed that glycosylated *CTSV* is present and that the higher band was indeed the glycosylated form of *CTSV*.

Yuki Niwa et al. observed that cathepsin V is glycosylated at both Asn^221^ and Asn^292^ in human fibrosarcoma HT1080 cells (24). To further investigate the glycan locus in lung cancer cells, we used liquid chromatography coupled with tandem mass spectrometry (LC-MS/MS) to determine certain sites of glycosylation for *CTSV*. Glycopeptides carrying N-glycans were identified for 2 N-glycosylation sites, which is consistent with a previous study (**Figures 2B, C**). The substitution of asparagine (N) to glutamine (Q) (N221Q, N292Q or both [2NQ]), caused a certain level of decrease in glycosylation when compared with that in the wild-type (WT) cells (**Figures 2D, E**). When treated with the N-linked glycosylation inhibitor tunicamycin, the glycosylation of *CTSV* was completely suppressed (**Figure 2F**). Similarly, hemagglutinin (HA)-tagged *CTSV* had a molecular weight shift from its regular size when treated with Endo H and PNGase F (**Figure 2G**). Together, the results suggested that *CTSV* is exclusively N-glycosylated at N221 and N292 in lung cancer cells.

### Glycosylation of *CTSV* Is Associated With Metastasis in Lung Cancer Patients

**Figure 3:**
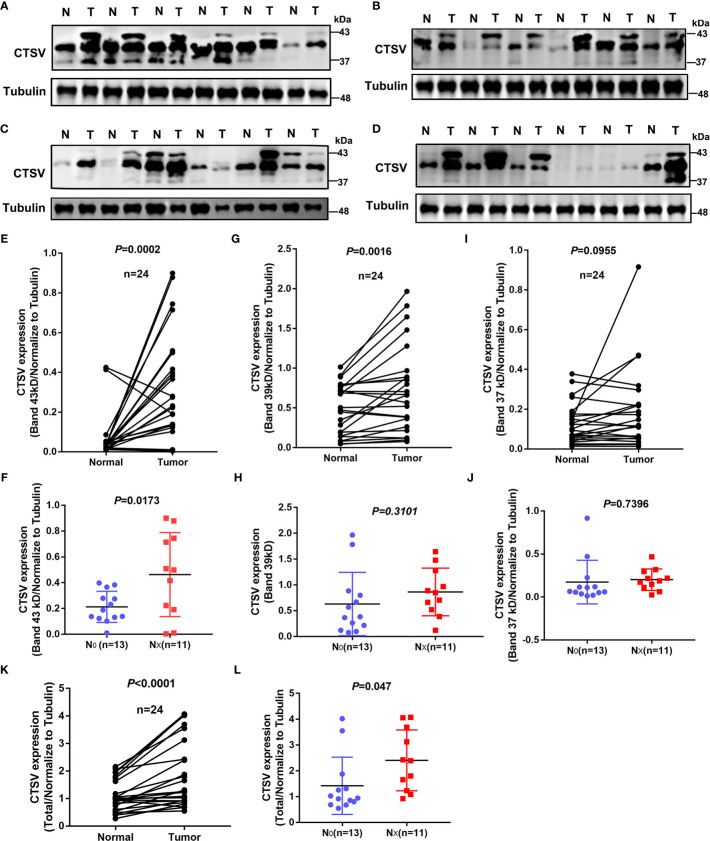
Glycosylation of *CTSV* is correlated with metastasis in lung cancer. **(A–D)** Western blot analysis of 24 pairs of lung specimens from lung cancer patients using anti-*CTSV* antibody. **(E–I, K)** Quantification of the Western blot bands by using ImageJ 6.0. **(F–J, L)** All patients were divided into the no lymph node metastasis group and the lymph node metastasis group, and *CTSV* expression was analyzed by Western blotting.

Next, we explored the clinical relevance of *CTSV* glycosylation in the lymph node metastasis of lung cancer. Western blot analysis of 24 paired lung samples showed that glycosylated *CTSV* was expressed at different levels in lung cancer tissues (**Figures 3A–D**). Importantly, three bands were observed in most lung cancer tissues but one or two bands were observed in normal tissues; therefore, we suspected that the glycosylation of *CTSV* might be associated with the progression of lung cancer. In this regard, we further assessed the relationship of glycosylation level with lymph node metastasis. Our data showed that the protein levels of the first and second bands of *CTSV* (the glycosylated forms of *CTSV*; bands at 43 and 39 kDa) were significantly higher in tumor tissues than in normal tissues, and no significant differences were observed in the third band (**Figures 3E, G, I**). In addition, we found that a higher protein level of the first glycosylation band (band 43 kDa) in lung cancer patients was correlated with lymph node metastasis (*P*=0.0173), while no significant changes were observed in the second and third bands (39 kDa and 37 kDa) of *CTSV* (**Figures 3F, H, J** and **Supplementary Table 1**). Furthermore, the data also showed that the protein level of total *CTSV* was significantly higher than that in normal tissues and was correlated with lymph node metastasis as well (**Figures 3K, I**). Together, the results suggested that the level of glycosylation CTSV (band 43 kDa) might be a more sensitive prognostic marker for lung cancer patients with metastasis.

### Glycosylation of *CTSV* Determine its Secretion and Metastasis of Lung Cancer Cells

**Figure 4:**
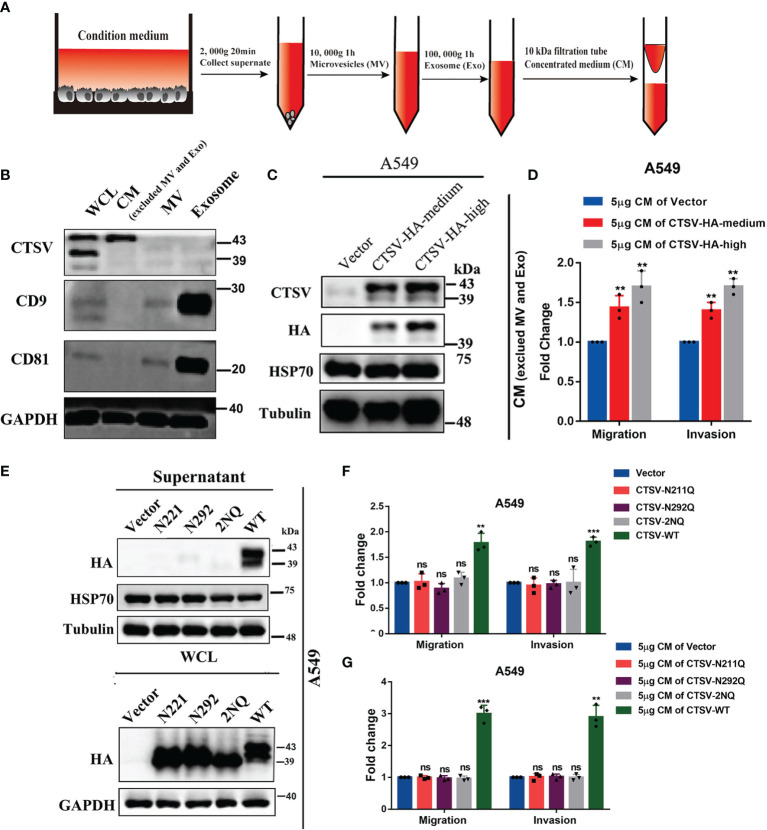
Mutation of glycosylation sites inhibits *CTSV* secretion and suppresses the metastasis of lung cancer cells. **(A)** Schematic diagrams of concentrated medium (CM) concentration. **(B)** Western blot analysis of CM using anti-*CTSV*, anti-CD9, anti-81 and anti-GAPDH antibodies. **(C)** Western blot analysis of the indicated stable A549 cells using anti-*CTSV*, anti-HA, anti-HSP70 and anti-Tubulin antibodies. **(D)** Migration and invasion assay of A549 cells pretreated with 5μg of *CTSV*-HA supernatant. **(E)** Western blot analysis of supernatant and whole cell lysate of A549 cells with N221Q, N292Q or both (2NQ) using anti-HA and anti-HSP70 antibodies. **(F)** Migration and invasion assay of A549 cells with N221Q, N292Q or both (2NQ). **(G)** Migration and invasion assay of A549 cells pretreated with 5μg of *CTSV*-N221Q, *CTSV*-N292Q or *CTSV*-2NQ supernatant. The results are shown as the mean ± SD of three biological independent experiments. **P < 0.01, ***P < 0.001 was analyzed by using the Student’s t-test. ns represent no significant change.

*CTSV* acts as an intracellular proteolytic enzyme, we suspected that *CTSV* is secreted from the cells to the intercellular space. To this end, we collected concentrated medium (CM) with a concentration column (10kDa) from the condition medium of indicated cells, which removed microvesicles (MV) and exosome (Exo), and Western blotting was conducted to determine the protein level of *CTSV* and exosome markers. As a result, we have excluded MV and Exo from condition medium, and 43 kDa *CTSV* was detected in CM, but not in MV and Exo (**Figures 4A, B**). Additionally, *CTSV* overexpression led to higher protein levels of *CTSV* in CM (**Figure 4C**). We then performed migration and invasion assays in A549 cells pretreated with 5μg CM of *CTSV*-HA-medium or *CTSV*-HA-high cells(Overexpression effect). We found that the CM of the indicated cells promoted the migration and invasion of A549 cells (**Figure 4D**).

Glycosylation is vital for maintaining protein structure and function. Secreted cathepsins have emerged as potent effectors that modify the tumor microenvironment by degrading the ECM (29). However, the role of N-glycosylated *CTSV* in determining its pro-metastatic behavior remains unclear. To determine the contribution of glycosylation to *CTSV* distribution, we performed Western blotting assays in supernatant and whole cell lysate and found that N221Q, N292Q and 2NQ suppressed the secretion of *CTSV* from intracellular to extracellular matrix (**Figure 4E**). Next, overexpression of *CTSV*-N221Q, *CTSV*-N292Q, *CTSV*-2NQ or wild type *CTSV* in A549 cells, respectively, we found N221Q, N292Q, both [2NQ] *CTSV* did not change the migration and invasion ability, but wild-type *CTSV* significantly increased the migration and invasion of A549 cells (**Figure 4F**). Consistently, we performed migration and invasion assays in A549 cells pretreated with 5μg CM of *CTSV*-N221Q, *CTSV*-N292Q, *CTSV*-2NQ, or *CTSV*-WT cells, the data revealed that CM from the *CTSV*-N221, *CTSV*-N292 or *CTSV*-2NQ cells showed no significant change in migration and invasion, but greatly promoted cell migration and invasion in wild-type CM group (**Figure 4G**). Interestingly, different from a previous study (24), we found that both glycosylation sites N221 and N292 of *CTSV*, rather than N292 alone, are important for its secretion, this regulating its promoting effect on tumor metastasis in lung cancer. Collectively, these results supported that glycosylation of *CTSV* is required for secretion, thereafter determine the metastasis behavior of lung cancer cells.

### Serum *CTSV* Distinguish Lung Cancer Patients From Healthy Donors

**Figure 5:**
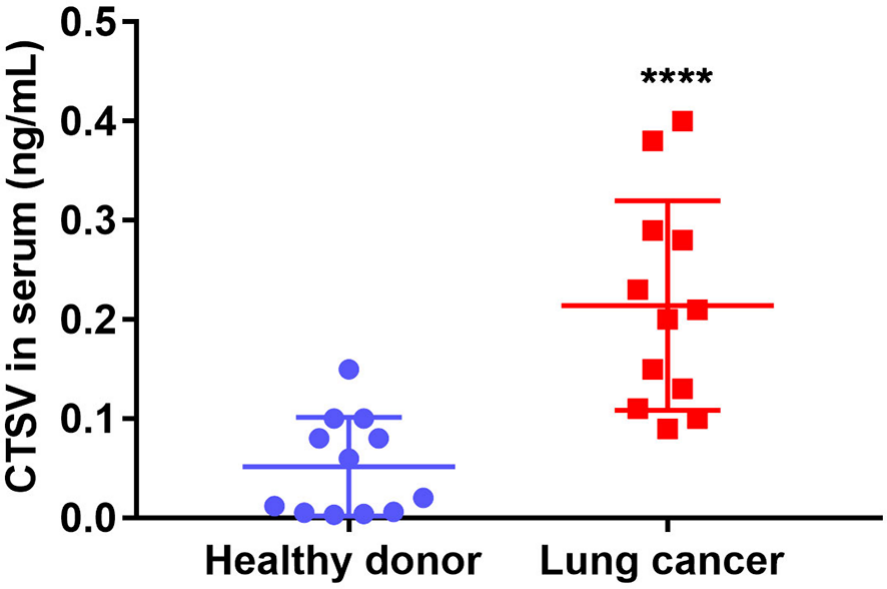
The level of *CTSV* on serum distinguishes patients with lung cancer from healthy donors. Elisa of human *CTSV* on serum in plasma samples from lung cancer patients and healthy donors. ****P < 0.0001 was analyzed by using the Student’s t-test.

Our previous evidence support that glycosylated *CTSV* (band 43 kDa) is particularly expressed in lung cancer samples, and the first glycosylation band (band 43 kDa) of *CTSV*, which has been secreted outside of the cells, we reasoned that the glycosylated *CTSV* might serve as a more sensitive prognosis marker for lung cancer. To this end, we isolated serum from the plasma of lung cancer patients and healthy donors, the level of *CTSV* in circulating serum was significantly higher in patients with lung cancer than that in healthy donors (**Figure 5**). Together, the data suggested that the level of *CTSV* in serum distinguished lung cancer patients from healthy donors, revealing that the level of *CTSV* in serum (glycosylation CTSV) might be a biological marker for lung cancer.

## Discussion

Cathepsins are vital acid proteolytic enzymes within lysosomes and therefore represent the crucial effector molecules of protein catabolism and autophagy (30, 31), which support the intense metabolic needs of proliferating cancer cells. In addition, cathepsins are integrated into almost all processes related to lysosomes, such as protein and lipid metabolism, protein degradation, antigen presentation, growth factor receptor cycling, stress signaling and lysosome-mediated cell death (29). They make critical contributions to cancer progression and dissemination in numerous cancer types.

Cysteine cathepsins are cysteine proteases that belong to the papain-like family; 11 cysteine cathepsins (B, C, F, H, K, L, O, S, V, W, and X) are encoded in the human genome (32). Cysteine cathepsins play crucial roles in regulating numerous physical processes, particularly proteolysis in the endolysosomal system and MHC II-mediated immune reactions (33, 34). Among others, these enzymes function differently in terms of localization and expression profile, tissue and cellular distribution, structural differences and modulation of enzyme activity. Although tightly controlled under physiological conditions, the dysregulation and overactivation of cathepsins in the extracellular milieu are marked hallmarks of numerous diseases, such as inflammation and inflammation-associated diseases, various types of cancer, bone disorders, rheumatoid arthritis and cardiovascular diseases (35–37). Therefore, cysteine cathepsins represent promising biological targets for therapeutic intervention.

Mutant *HRAS* upregulates both *CTSB* and *CTSL* and transforms mammary epithelial cells (38). *CTSS* expression in cancer cells is crucial for the promotion of colorectal cancer growth and progression (39). *CTSZ* promotes pancreatic neuroendocrine tumor growth and progression from both cancer cell-derived and tumor-associated macrophage-derived sources (40). However, the molecular mechanisms of cathepsins that lead to the malignancy of tumors remain unclear, which is amplified by the fact that members of the cathepsin superfamily have different roles in different types of cancer. Herein, our clinical data, based on RNA-seq, TCGA and GEO datasets, IHC and prognosis analysis, revealed that *CTSV* is a prognostic biomarker that is upregulated in lung cancer. The functional impact and mechanism of the dysregulation of *CTSV* in lung cancer remain to be determined.

N-glycosylation plays an important role in determining the structure and functions of proteins (41). The glycosylation of *CTSV* has been reported before in human fibrosarcoma HT1080 cells (24). Consistently, we showed that glycosylation of *CTSV* generally exists in lung cancer tissues and lung cancer cell lines, and protein mass spectrometry identified glycosylation at the N221 and N292 residues. Mutation of the N221 and N292 residues with Q residues, generating N221Q and N292Q, decreased the glycosylation of *CTSV*, thereby inhibited the secretion of *CTSV*, and remarkably suppressed the metastasis, invasion and migration of lung cancer cells. Importantly, we found that the glycosylation band (43 kDa) was positively correlated with lymph node metastasis, which suggests that the glycosylation of *CTSV* can serve as a risk factor for malignant progression of lung cancer. To further understand the role of *CTSV* in lung cancer, it is critical to dissect the complex processes by which upregulation of protease expression disrupts the homeostasis of normal tissue. The results that the CM of *CTSV* overexpression cells presented a stronger band confirmed our hypothesis. Our data provide mechanistic insight into this metastasis-driven issue.

In conclusion, our research showed that *CTSV* is upregulated in lung cancer and correlated with a poor prognosis, indicating that *CTSV* might be a prognostic biomarker for lung cancer. Herein, we confirmed that *CTSV* is N-glycosylated at N221 and N292, and its pro-metastatic behavior is glycosylation and extracellular secretion dependent. Notably, the level of *CTSV* in serum distinguished lung cancer patients from healthy donors and the glycosylated 43 kDa *CTSV* (secreted *CTSV*) is associated with lymph node metastasis. Collectively, our findings suggest that glycosylated *CTSV* could be a sensitive biomarker for lung cancer patients with metastasis.

## Data Availability Statement

The datasets presented in this study can be found in online repositories. The names of the repository/repositories and accession number(s) can be found in the article/**Supplementary Material**.

## Ethics Statement

The studies involving human participants were reviewed and approved by Ethics Committee of Sun Yat-sen University Cancer Center. The patients/participants provided their written informed consent to participate in this study.

## Author Contributions

LY: Conceptualization, Methodology, Writing-Original draft preparation. QZ, YD: Methodology, Data curation, Software, Formal analysis. YQ: Visualization, Investigation. WY, YL: Resources, Validation, Supervision. LY, WY, YL: Writing,Reviewing and Editing. All authors contributed to the article and approved the submitted version.

## Conflict of Interest

The authors declare that the research was conducted in the absence of any commercial or financial relationships that could be construed as a potential conflict of interest.

## Publisher’s Note

All claims expressed in this article are solely those of the authors and do not necessarily represent those of their affiliated organizations, or those of the publisher, the editors and the reviewers. Any product that may be evaluated in this article, or claim that may be made by its manufacturer, is not guaranteed or endorsed by the publisher.

## Abbreviations

*CTSC*, cathepsin C; *CTSF*, cathepsin F; *CTSH*, cathepsin H; *CTSO*, cathepsin O; *CTSV*, cathepsin V; *CTSW*, Cathepsin W; CM, Concentration medium; ECM, extracellular matrix; Endo H, endoglycosidase; Exo, exosome; HA, hemagglutinin; IHC, Immunohistochemistry staining; MS, Protein mass spectrometry; MV, microvesicles.
