# Supplementary material for: Glycosylated Cathepsin V Serves as a Prognostic Marker in Lung Cancer
Source: Front Oncol. 2022 Apr 13;12:876245. doi: 10.3389/fonc.2022.876245 (PMC9043764; doi:10.3389/fonc.2022.876245)
Supplement: Supplementary file 1 [file DataSheet_1.docx]

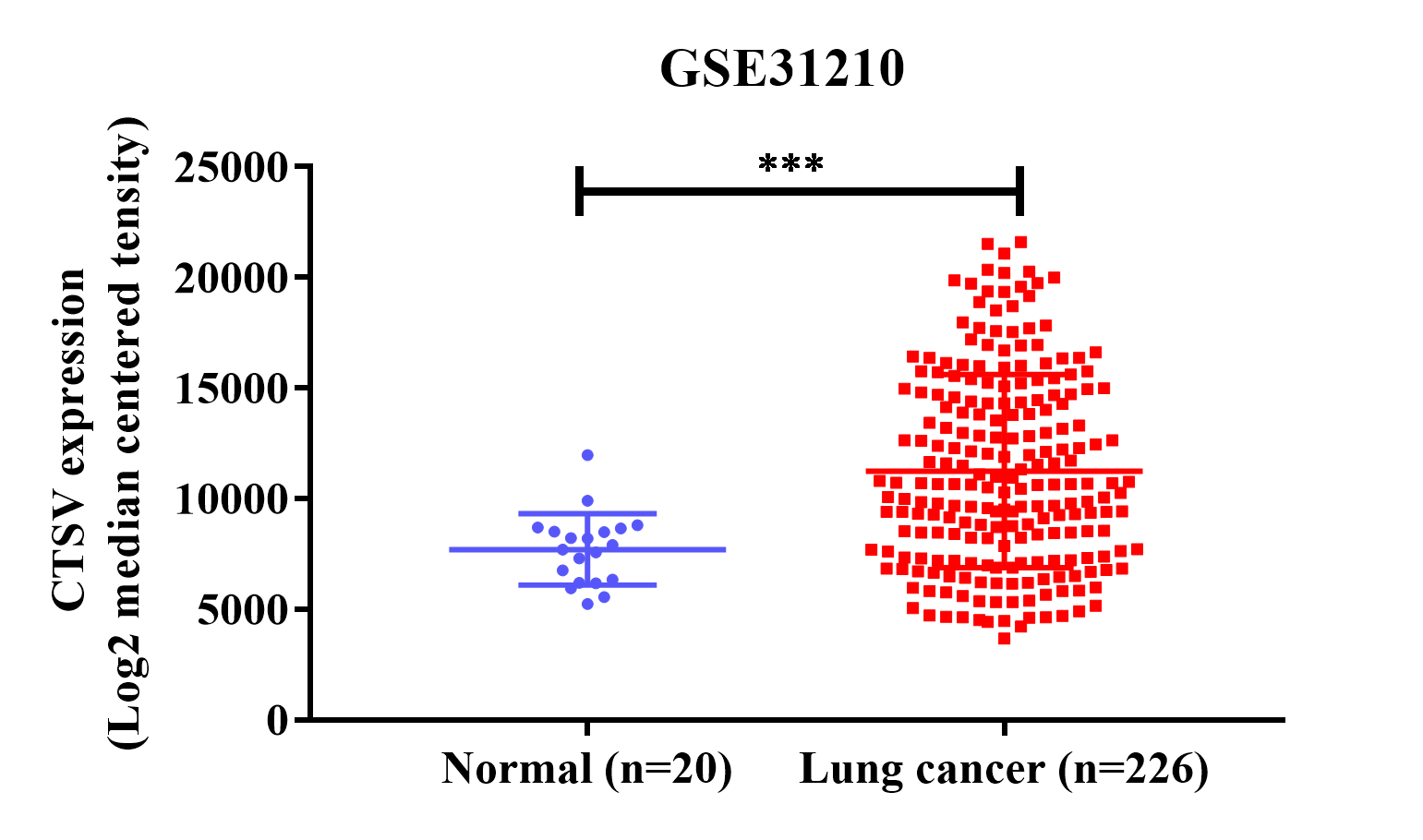

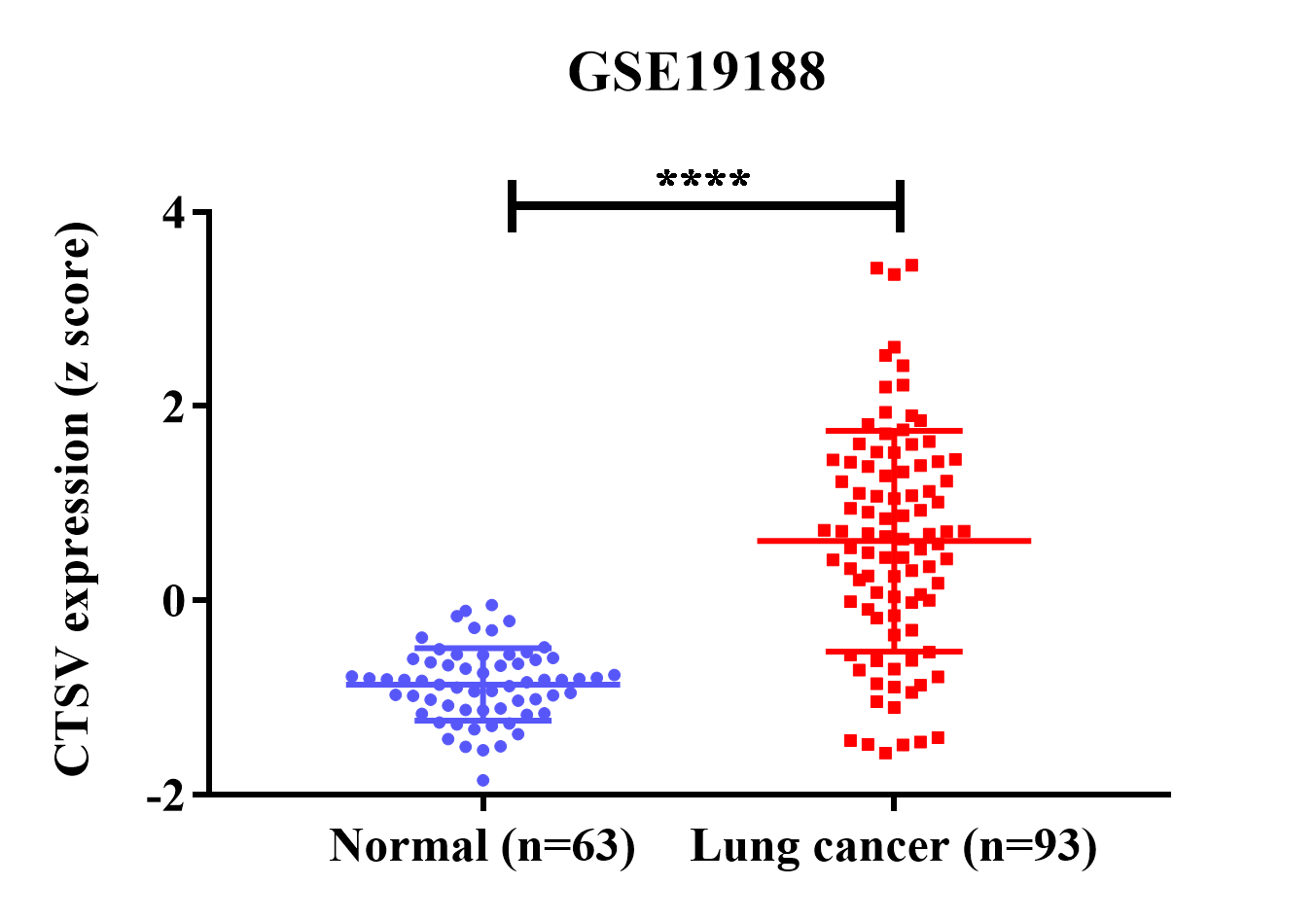


c

a

b


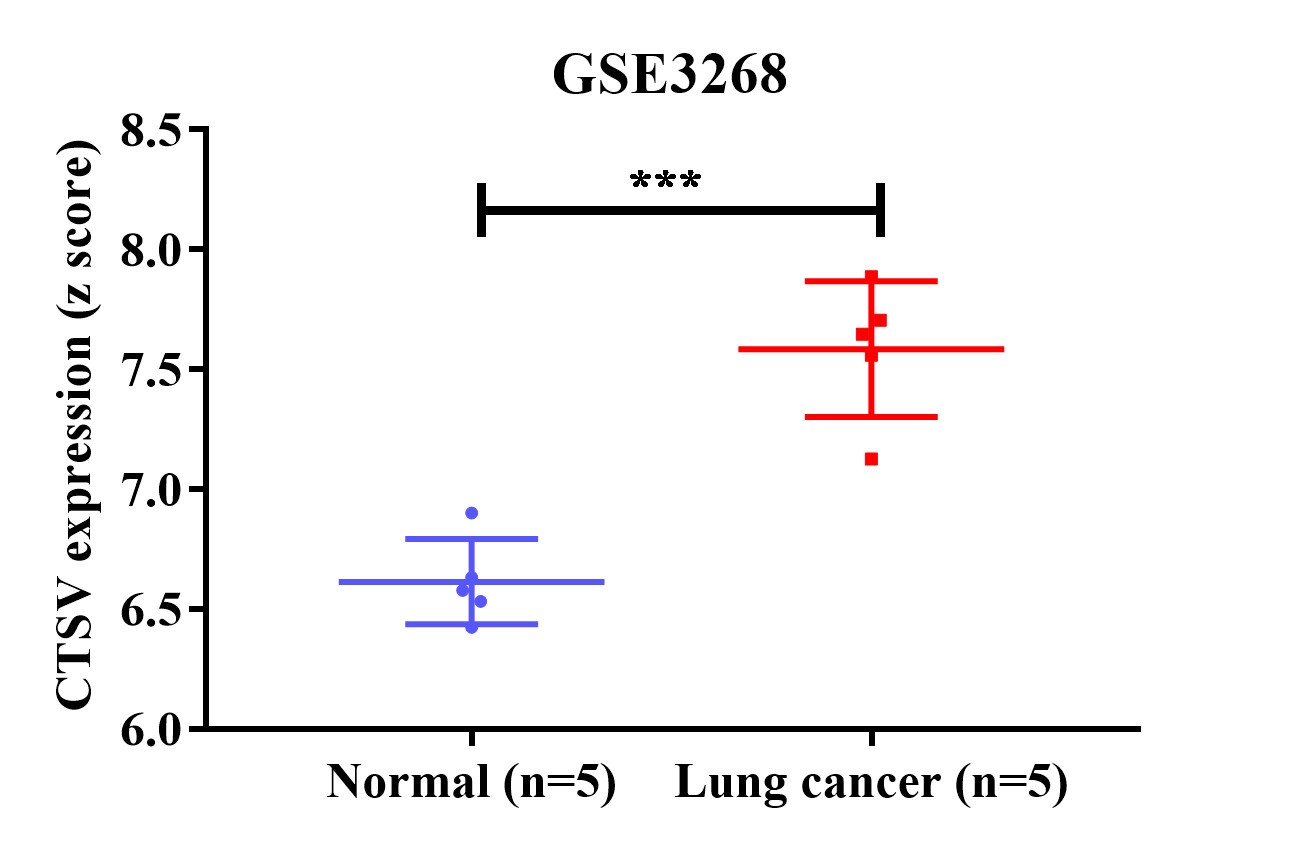


**Figure S1 CTSV is overexpressed in lung cancer**

1. c) Relative expression of CTSV in normal and lung cancer tissue samples from GEO database.
